# Novel *ADPRS* Missense Variant (p.Leu162Pro) Causes Stress-Induced Childhood-Onset Neurodegeneration With Ataxia and Seizures

**DOI:** 10.1212/NXG.0000000000200375

**Published:** 2026-04-17

**Authors:** Priscilla Doria de Mattos, Rafael Dias de Moura, Murilo Fígaro Bertolino, Penélope Ferreira Valente, Isaac Araujo Matos, Vitor Marques Caldas, Paulo Emidio Lobao Cunha, Fernando Kok, Nicolas Carlos Hoch

**Affiliations:** 1 Department of Biochemistry, Chemistry Institute, University of São Paulo, Brazil;; 2 Instituto Ranvier, Brasília, Brazil;; 3 Medical School, University of Brasília, Brazil;; 4 Department of Pediatric Neurology, Medical School, University of Sao Paulo, Brazil; and; 5 Mendelics Análise Genômica, São Paulo, Brazil.

## Abstract

**Background and Objectives:**

ADP-ribosylation is a post-translational modification critical for DNA repair, chromatin remodeling, and cellular stress responses. The enzyme ARH3/ADPRHL2 (encoded by the *ADPRS* gene) is a member of the ADP-ribosyl-acceptor hydrolase family and plays a pivotal role in the removal of mono-ADP-ribosylation, particularly in response to DNA damage. Variants in the *ADPRS* gene cause *stress-induced childhood-onset neurodegeneration with variable ataxia and seizures* (CONDSIAS). Here, we present the case of an 11-year-old Brazilian girl with compound heterozygous variants in *ADPRS*, including a novel missense variant, c.485T>C; p.Leu162Pro, of unknown clinical significance. This study aimed to determine the pathogenicity of this variant and to test the molecular rationale of using minocycline as a therapeutic strategy for CONDSIAS cases.

**Methods:**

Clinical identification and diagnosis of the patient was based primarily on MRI and whole exome sequencing. Functional assays were performed in patient fibroblast cell lines using Western blotting and immunofluorescence-based assays and compared with an ARH3 KO cell line generated by CRISPR/Cas9 technology.

**Results:**

Our data indicate that the novel p.Leu162Pro variant, combined with a known nonsense variant, c.316C>T; p.Gln106*, causes a severe reduction of ARH3 protein levels in patient fibroblasts. This leads to defective ARH3-dependent removal of ADP-ribosylation, both under basal conditions and following oxidative stress. Our results using minocycline indicate that it is a poor PARP1 inhibitor and was ineffective in reducing basal ADP-ribosylation in ARH3-deficient cells.

**Discussion:**

This study contributes to the growing body of knowledge on ARH3 deficiency associated with CONDSIAS, by identifying a new pathogenic variant and questioning the molecular rationale underlying the therapeutic use of minocycline in patients with CONDSIAS.

## Introduction

ADP-ribosylation (ADPr) is the transfer of the ADP-ribosyl group from NAD^+^ molecules to target proteins or nucleic acids, with the release of nicotinamide.^1,2^ In mammals, the enzymes responsible for intracellular ADP-ribosylation belong to the diphtheria toxin-like ADP-ribosyltransferase (ARTD) family, whose members are known as poly-ADP-ribose polymerases (PARPs) and tankyrases.^3^ Protein ADP-ribosylation plays key roles in signaling DNA strand breaks, which can arise from oxidative DNA damage caused by reactive oxygen or nitrogen species, as intermediates of cellular DNA repair pathways such as base excision repair (BER), or as part of DNA metabolism, such as topoisomerase activity and Okazaki fragment processing.^4,5^ PARP1 and PARP2 have high affinity for these DNA strand breaks and, once bound, catalyze the ADP-ribosylation of target chromatin proteins surrounding the DNA damage site, mainly histones and PARP1/2 themselves. This modification can be directed to the hydroxyl group of serine residues when PARP1 or PARP2 are in complex with HPF1 or be targeted to glutamate and aspartate residues in the absence of HPF1.^6^ The resulting poly-ADP-ribose (PAR) chains recruit DNA repair factors such as XRCC1, accelerating DNA repair.^3,7^

After completion of DNA repair, the timely removal of ADP-ribose modifications from proteins is important to restore chromatin structure and is mainly catalyzed by poly-ADP-ribose glycohydrolase (PARG) and ADP-ribosyl hydrolase 3 (ARH3).^8^ PARG cleaves the bonds between ADP-ribose residues and is the main enzyme responsible for degrading PAR chains.^9^ In addition, PARG was recently shown to hydrolyze the ester linkage between mono-ADP-ribose and glutamate or aspartate residues, indicating it can single-handedly hydrolyze Glu/Asp-linked PAR chains.^10^ However, PARG is unable to cleave mono-ADP-ribose linkages to serine residues, which instead relies on ARH3/ADPRHL2 (encoded by the *ADPRS* gene),^11^ making ARH3 a central factor for the complete removal of DNA damage-induced ADPr and the maintenance of the chromatin landscape in situations of genotoxic stress.

Stress-induced childhood-onset neurodegeneration with variable ataxia and seizures (CONDSIAS, OMIM#618170) is an autosomal recessive disease caused by variants in the *ADPRS* gene.^12,13^ Its symptoms vary, but include cerebral, cerebellar and spinal cord atrophy, developmental delay, cognitive impairments, stress-induced seizures, ataxia, axonal neuropathy, respiratory insufficiency, and sensory neuron deafness.^14-18^ The connection between loss of ARH3 function and neurodegenerative disorders is incompletely understood at a molecular level, but there is evidence that, in the absence of ARH3 activity, mono-ADP-ribose modifications accumulate on histones and impact transcriptional regulation.^19,20^

Here, we report the case of a Brazilian patient carrying compound heterozygous variants in *ADPRS*, including a novel missense variant which we show here to affect ARH3 protein stability, leading to functional ARH3 deficiency in patient fibroblasts. Because some patients with CONDSIAS are reported to be making use of minocycline,^17,21^ a tetracycline antibiotic that has been reported to inhibit PARP1 activity,^22^ potential effects of minocycline on PARP1 activity were also investigated.

## Methods

### Genetic Analysis

DNA was extracted from a peripheral blood sample, then exome sequencing was performed by Mendelics (São Paulo, Brazil) according to the company's workflow, using NovaSeq 6000 System (Illumina®) and proprietary analysis software Abracadabra®.

### Molecular Modelling

To model the structural consequences of the ARH3-Leu162Pro substitution, the ARH3 crystal structure PDB 5ZQY, with a resolution of 1.58 Å, was used.^23^ The L162P substitution was generated by Dumbrack rotamer library^24^ in Chimera^25^ using the same crystal structure. The impact of the L162P substitution on the global ARH3 structure was modeled using AlphaFold3.^26^ Figures were generated using Pymol 1.8.

### Cell Culture

All cell lines were grown at 37°C in 5% CO_2_. Primary fibroblasts were obtained from skin biopsies and grown in MEM (Thermo Fisher, MA). hTERT-RPE1 cells (ATCC: CRL-4000) were maintained in DMEM/F-12 (Thermo Fisher, MA) containing 15 mM HEPES (Merck, NJ). Media were also supplemented with 10% fetal bovine serum (Thermo Fisher, MA) and penicillin/streptomycin 100 U/mL/100 μg/mL (Thermo Fisher, MA).

### Generation of Knockout Cell Line

Guide RNA (gRNA) targeting the *ADPRS* gene was designed using Benchling.^27^ Sequences for the gRNA were 5′-CACCGATGGCGCCATTGTAACCCA-3’ (forward) and 5′-AAACTGGGTTACAATGGCGCCATC-3’ (reverse). These oligonucleotides were annealed and inserted into eSpCas9(1.1) vector^28^ (addgene #71814) using restriction enzyme BbsI and T4 DNA ligase from NEB (MA). Competent *Escherichia coli* cells (NEB® 5-alpha strain, MA) were transformed by heat shock and cloned vectors were purified by the Monarch® Plasmid Miniprep Kit (NEB, MA). hTERT-RPE1 cells were then transfected with this vector by electroporation, using Neon™ Transfection System (Thermo Fisher, MA), along with vector pCD2E (a kind gift of K. Caldecott, Univ. Sussex, UK), which confers resistance to G418. Cell populations were grown for 5 days in medium containing 1 mg/mL G418 for selection of transfected cells. Individual clones were isolated from this population and screened for ARH3 protein levels by Western blotting. Genomic DNA was extracted from selected clones, and the edited locus was amplified by PCR and Sanger sequenced using BigDye Terminator 3.1 (Thermo Fisher, MA). Sequencing results were visualized with SnapGene Viewer.

### Cell Treatments

Olaparib (Selleckchem, Houston, TX) and minocycline (Sigma-Aldrich, MO) were both used at a final concentration of 10 µM. Treatment with H_2_O_2_ was performed in PBS, followed by washing with PBS and incubation in growth medium, as indicated. MG-132 was used at 5 µM for 6 hours.

### Antibodies

Pan-ADP-ribose antibody-like detection reagent (MABE1016, Millipore, MA),^29^ HRP-conjugated mono-ADP-Ribose antibody-like detection reagent clone AbD43647 (TZA020P, Bio-Rad, CA),^30^ anti-ADPRHL2(ARH3) antibody (HPA027104, Sigma-Aldrich, MO); anti-Ubiquitin (P37) Antibody (#58395, Cell Signaling, MA); anti-alpha Tubulin antibody (ab18251, Abcam, Cambridge, UK).

### Western Blotting and Quantification

Western blotting was performed essentially as previously described.^31,32^ Cells were washed with PBS and lysed by adding incomplete Laemmli buffer (without bromophenol blue or DTT/beta-mercaptoethanol) at 100°C directly to the well and scraping. Lysates were transferred to microtubes, then boiled at 100°C for 15 minutes. Protein concentrations were determined using BCA Protein Assay kit (Pierce, WI). After adjusting concentration, beta-mercaptoethanol (5% v/v) and bromophenol blue were added, then samples were boiled again. For each sample, 15–40 µg of protein were loaded into SDS-PAGE gels, then transferred to nitrocellulose membranes (Bio-Rad, CA). Membranes were stained with Ponceau S (Sigma-Aldrich, MO) for visualization and loading control. Some membranes were cut to isolate different molecular weight regions, allowing for simultaneous use of multiple antibodies. Membranes were destained with TBS-T and blocked with 5% skimmed milk or 5% bovine serum albumin (BSA - Thermo Fisher, MA). Then, membranes were incubated with primary antibodies overnight at 4°C (or 30 minutes at room temperature for tubulin). After multiple rounds of washing with TBS-T, HRP-conjugated secondary antibodies (Sigma-Aldrich, MO) were added, and after 1 hour of incubation at room temperature, membranes were washed again and then incubated with ECL Prime (Amersham, IL) reagent. For the mono-ADPr detection reagent, which is already HRP-conjugated, a single round of antibody incubation for 1 hour was followed by washing in TBS-T. Chemiluminescence signal was detected using a ChemiDoc MP Imaging System (Bio-Rad, CA) and quantified using ImageLab software (Bio-Rad, CA).

For experiments with patient fibroblasts and ARH3 KO cells, 2 types of quantification were performed: One using the whole lane and another in which the combined signal of the 2 strongest bands in the low molecular weight region, which we assume to be histones, were quantified. For minocycline experiments, total lane signal was used. To correct for loading variations, these values were divided by the total colorimetric Ponceau S signal of the corresponding samples. These ADPr/Ponceau ratios were then normalized relative to the H_2_O_2_-treated WT RPE1 (for RPE1 cells) or H_2_O_2_-treated 1BR (for fibroblasts), which were arbitrarily assigned a value of 1. This normalization to the treated samples (rather than untreated controls) was necessary to reduce spurious variation between replicate experiments because of the low signal in untreated samples.

### Immunofluorescence Staining

Immunofluorescence staining was performed essentially as described in.^31^ Cells were seeded on 96-well microplates with optical polymer base (Thermo Fisher, MA). After treatment, cells were washed with PBS and fixed with 4% PFA (EMS, PA). Cells were then permeabilized with 0.2% TritonX-100 in PBS, blocked in 10% fetal bovine serum in PBS for 1 hour, then incubated with primary antibody (diluted 1:1000 in blocking solution) for 1 hour in a humid environment at room temperature. After washing with PBS, samples were incubated with fluorescently labeled secondary antibodies (Thermo Fisher, MA) (diluted 1:1000 in blocking solution) for 1 hour at room temperature, washed again and stained with 0.2 µg/mL DAPI dissolved in PBS. Cells were kept in glycerol and refrigerated until image acquisition.

### Imaging and Analysis

For quantitative fluorescence microscopy analyses, images were acquired on a customized TissueFAXS i-Fluo system (TissueGnostics, Wien, Austria) mounted on a Zeiss Axio Observer 7 microscope (Zeiss, Oberkochen, Germany), using 20 × Plan-Neofluar (NA 0.5) objective and an ORCA Flash 4.0 v3 camera (Hamamatsu, Shizuoka, Japan). Images were acquired using automated autofocus settings and were analyzed using StrataQuest software (TissueGnostics, Wien, Austria).^32^

For each sample, 25 fields of view were acquired, totaling thousands of cells per replicate sample. Individual nuclei were detected in the DAPI channel and gated to exclude imperfectly detected events and artifacts, using DAPI signal intensity and nuclear compactness. This nuclear mask was then used to quantify the mean intensity ADP-ribose signal within each nucleus from background-subtracted images. The signal intensity from thousands of nuclei per well were averaged to produce a single mean intensity value for each sample, and these values were then normalized to the treated control sample, which was arbitrarily assigned a value of 1.

### Statistical Analysis

All experiments were performed independently at least 3 times, unless otherwise stated in the figure legend. All graphs and statistical analyses were generated using GraphPad Prism 8.0.1 software, here displayed with the individual data points and their mean ± SEM. Values were normalized relative to a control condition (indicated in the figure legend) for each replicate, considered as 1. Statistical comparisons between samples were performed using one-way ANOVA with the Tukey multiple comparisons test and shown as ns = not significant, * = *p* < 0.05, ** = *p* < 0.005, *** = *p* < 0.0005, and **** = *p* < 0.0001.

### Standard Protocol Approvals, Registrations, and Patient Consents

The patient's family agreed to the collection of biopsies and their usage for this study by signing free and informed consent terms which were submitted to the research ethics committee of Instituto de Biociências–Universidade de São Paulo (CEP-IBUSP). This study was thus approved by CEP-IBUSP under the registration (CAAE) number 39085820.2.0000.5464, which can be verified at Plataforma Brasil.^33^

### Data Availability

Anonymized data not published within this article will be made available by request from any qualified investigator.

## Results

### Case Report

The patient described here is a female, currently 11 years old, with manifestations beginning in the first year of life, including focal motor seizures, cerebellar ataxia, intermittent cervical dystonia, and ptosis. By the age of 4, the patient developed neurogenic bladder and paralytic ileus (requiring ileostomy), demyelinating polyneuropathy, speech deterioration, and subtle occipital and cerebellar atrophy identified in MRI, a finding not present in 2 prior examinations performed at ages 2 and 3. The patient evolved with continuous invasive ventilatory support and the clinical manifestations worsened progressively until the age of 6, often exacerbated by infections, with multiple hospitalizations.

In the early years of life, the condition was interpreted as autoimmune encephalitis (anti-GAD encephalitis), with a good response to immunosuppressive treatments, including IV immunoglobulin and rituximab. However, the autoimmune hypothesis was later discarded based on new serum and CSF analyses. Immunologic treatment was discontinued after genetic diagnosis of ARH3 deficiency (described below). After age 7, the patient evolved with clinical stability, although occasional exacerbations have been observed, particularly associated with respiratory infections or temperature fluctuations. However, no hospitalizations occurred because of clinical complications, epileptic seizures were stabilized with the use of levetiracetam and clobazam in high doses, and the gastrointestinal condition remains stable with nutritional support and ileostomy care.

In addition to the neurologic and gastrointestinal manifestations, and after stabilization of the underlying medical condition, the patient was diagnosed with autism spectrum disorder (ASD), secondary to persistent difficulties in social interaction, restricted communication, and typical repetitive and restricted behaviors of the disorder. The introduction of augmentative and alternative communication through tablet allowed for better expression of the patient's needs and feelings, significantly improving quality of life and social integration. Minocycline was introduced at age 9, and the patient has been on continuous treatment since then, showing no adverse effects, but with limited assessment of its effectiveness, because clinical stability occurred before its introduction. Currently, the patient attends school on a reduced schedule, is off mechanical ventilation during wakefulness, and has adapted well to therapeutic changes. The patient's clinical evolution, particularly the stabilization of neurologic conditions and improvement in social interaction, suggests a favorable prognosis, with effective symptom control and a successful multidisciplinary therapeutic approach.

### A Novel Missense Variant in *ADPRS*

**Figure 1:**
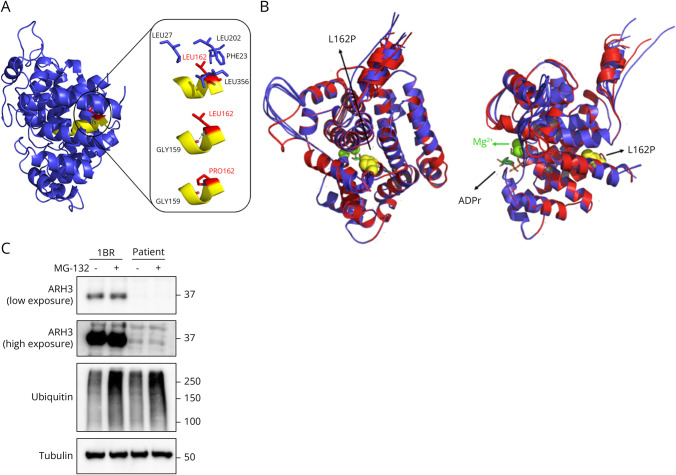
Leu162Pro Impacts ARH3 Protein Stability (A) Molecular model of WT ARH3 (PDB 5ZQY) modified to show the Leu162Pro substitution in red. The hydrophobic interactions of Leu162 with surrounding residues Leu27, Leu202, Phe23, and Leu356 (top right) and the loss of a backbone hydrogen bond between Leu162 and Gly159 (middle and bottom right) are shown. (B) Superimposed Alphafold3 models of the WT ARH3 protein (red) and of the ARH3-L162P variant (blue). ADP-ribose is shown as sticks, magnesium atoms as green spheres. The 5 best models for each sequence are shown. (C) Immunoblots for ARH3 at low and high exposures (top), ubiquitin (middle), and tubulin (bottom) in 1BR controls and patient fibroblasts, treated or not with 5 µM MG-132 for 6 hours.

Genetic testing was performed on patient material at Mendelics (São Paulo, Brazil), a commercial clinical genomics facility, by whole-exome Illumina sequencing and revealed compound heterozygous variants in the *ADPRS* gene, which encodes ARH3. One allele, confirmed to be paternally inherited, contained a known variant (NM_017825.3 c.316C>T; p.Gln106*),^12^ which can be classified as “pathogenic” according to ACMG/AMP criteria,^34^ while the maternally inherited allele was found to contain a novel missense variant (NM_017825.3 c.485T>C; p.Leu162Pro), classified as “uncertain significance” at the start of this study. The latter variant is not found in gnomAD or ClinVar (Table) and was predicted to be “probably damaging” by PolyPhen-2 because Leu162 is a highly conserved residue. To explore the molecular consequences of this variant further, we modeled the structural consequences of the L162P substitution, which revealed that L162 occurs within an alpha-helix and is buried in a hydrophobic pocket (Figure 1A). Both the hydrophobic interactions with surrounding residues and a helix-stabilizing backbone hydrogen bond with Gly159 are predicted to be lost with the proline substitution (Figure 1A). A comparison of the Alphafold3 models of the WT and L162P proteins revealed substantial changes in several additional loops and helices throughout the protein, indicating that the overall protein structure could be affected by the variant (Figure 1B). These data, combined with the patient's characteristic clinical presentation, led us to hypothesize that L162P is a loss-of-function variant.

**Table:** Exome Sequencing Results

Gene	Genomic position (GRCh37) and variation	cDNA change	Consequence	No. of copies	Allele frequency (gnomAD)
ADPRS	chr1.36.557.226 C>T	c.316C>T (NM_017825.3)	p.Gln106*	1	0.000003132
ADPRS	chr1.36.557.395 T>C	c.485T>C (NM_017825.3)	p.Leu162Pro	1	Variant not found

Whole-exome sequencing results identified compound heterozygous variants in the gene encoding ARH3. The predicted consequences of the variants on the protein products are shown. Allele frequency was obtained from the gnomAD database.^52^

### L162P Substitution Destabilizes ARH3 Protein

Next, we established a fibroblast cell line from a patient skin biopsy and performed Western blotting to determine ARH3 protein levels in these cells. Given that the anti-ARH3 antibody (HPA027104) used for this experiment was raised against amino acids 206-266 of ARH3, any truncated protein produced from the Q106* allele is not expected to be detected by this reagent, while the L162P substitution should not affect antibody recognition, allowing determination of protein levels expressed from this allele only. This experiment revealed a profound reduction in anti-ARH3 signal in patient fibroblasts compared with controls, such that ARH3 protein was only weakly detectable in patient samples with long exposure times (Figure 1C). Therefore, this result indicates that the novel L162P substitution is indeed pathogenic, by strongly destabilizing ARH3 protein.

We hypothesized that the strong reduction in ARH3-L162P levels could be the consequence of proteasomal degradation of the variant protein. To test this hypothesis, cells were treated with the proteasome inhibitor MG-132 (Figure 1C), which, as expected, led to the accumulation of poly-ubiquitylated proteins. However, ARH3-L162P protein levels were not affected by MG-132 treatment, indicating that variant protein degradation occurs by other mechanisms that are yet to be determined.

### Functional ARH3 Deficiency in Patient Fibroblasts

Hydrogen peroxide (H_2_O_2_) is known to induce oxidative DNA damage, including the formation of single-stranded DNA breaks that trigger the recruitment and activation of PARP1 and PARP2.^35^ This leads to poly-ADP-ribosylation of chromatin proteins, which is subsequently reversed by the combined activities of PARG and ARH3. Therefore, ARH3 deficiency leads to defects in the removal of peroxide-induced ADP-ribosylation.

**Figure 2:**
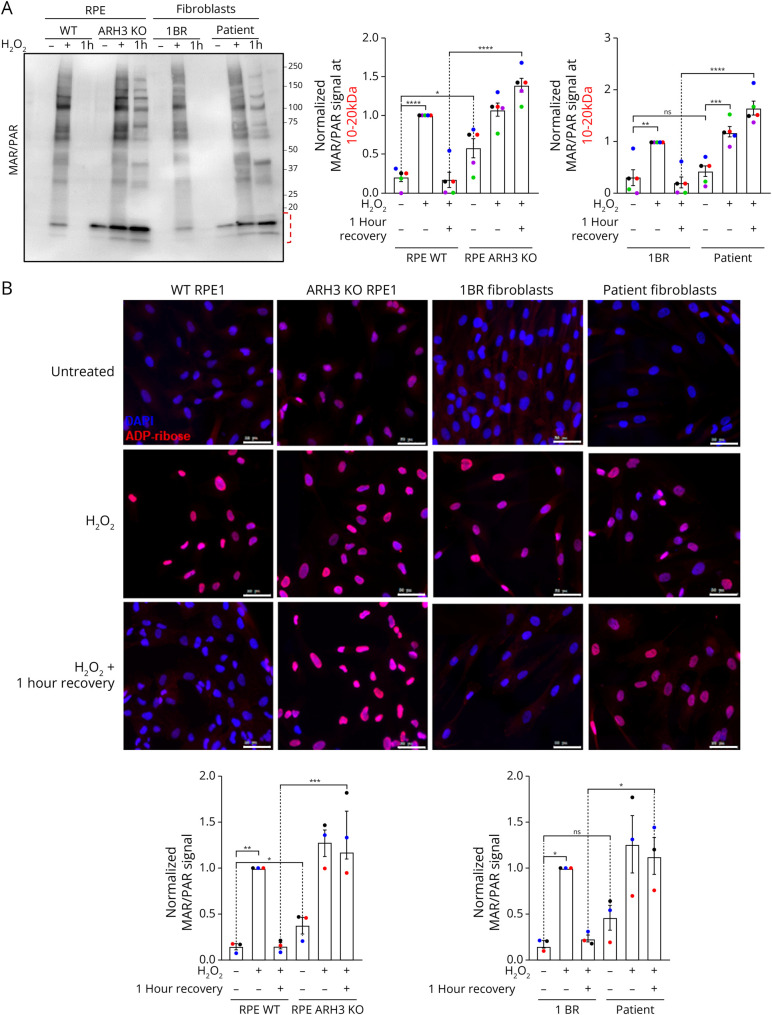
Patient Fibroblasts Accumulate ADP-Ribosylated Proteins (A) Representative image (left) and quantification of signals in the 10–20 kDa range (right) of MAR/PAR immunoblots using pan-ADP-ribose (MABE1016) reagent, in RPE1 WT cells, ARH3 KO cells, control fibroblasts (1BR), or patient fibroblast untreated (−), treated with 600 µM H_2_O_2_ for 10 minutes (+), or treated with 600 µM H_2_O_2_ for 10 minutes, washed and allowed to recover in medium for 60 minutes (1 hour). The dotted lines below the 20 kDa marker indicate the region of the membrane used for quantification. Quantification of the full lane is shown in eFigure 2A (B) Representative images (top) and quantification (bottom) of pan-ADP-ribose (MAR/PAR) immunofluorescence assay after the same treatment conditions; scale bars = 50 µm. Nuclei were stained with DAPI (blue) or pan-ADP-ribose (MAR/PAR) reagent (red). The mean MAR/PAR intensity within each nucleus was quantified, and the values for thousands of nuclei were averaged for each replicate/sample. Graphs show mean and SEM of 5 (A) and 3 (B) independent experiments. For normalization, the signal of WT RPE1 or 1BR cells treated with H_2_O_2_ in each replicate was arbitrarily defined as 1. The one-way ANOVA and Tukey test were performed. ns = not significant, * = *p* < 0.05, ** = *p* < 0.005, *** = *p* < 0.0005, and **** = *p* < 0.0001.

To study ARH3 functionality in patient fibroblasts, we first generated an hTERT-RPE1 cell line in which the *ADPRS* gene was knocked out by CRISPR-Cas9 technology (eFigure 1), to serve as a control for functional assays (referred to as “ARH3 KO” from here onwards). Next, we treated cells with hydrogen peroxide and measured the induction and removal of protein ADP-ribosylation either by Western blotting (Figure 2A) or by immunofluorescence microscopy (Figure 2B), using an antibody-like reagent that detects both mono-ADPr (MAR) and poly-ADPr (PAR) (MABE1016).^29^

As expected, hydrogen peroxide treatment strongly induced ADP-ribosylation 15 minutes after peroxide treatment, and this signal was almost completely removed within 1 hour of recovery in full growth medium (Figure 2A). As previously reported,^19^ ARH3 KO caused the accumulation of ADP-ribosylated proteins in the 10–20 kDa range, even before treatment (Figure 2A; lane 4 vs lane 1), likely reflecting defective removal of mono-ADP-ribose modification of histones arising from spontaneous DNA damage. A similar, but not statistically significant, effect was also observed in untreated patient fibroblasts compared with controls (Figure 2A; lane 10 vs lane 7; 10–20 kDa range), suggesting ARH3 deficiency in patient cells. On DNA damage induction with hydrogen peroxide, ARH3 KO cells displayed a substantial delay in ADPr hydrolysis on several proteins throughout the molecular weight range, again with a more prominent defect on removal of histone ADPr (Figure 2A, lane 6 vs lane 3). Similarly, defective hydrolysis of ADPr modifications, especially on histones, was also evident in patient fibroblasts compared with controls (Figure 2A, lane 12 vs lane 9), again indicating that these cells are deficient in ARH3 activity. While the quantification of MAR/PAR signal specifically in the 10–20 kDa range (Figure 2A) most strongly reflected ARH3 functionality, quantification of MAR/PAR signal across the whole molecular weight range revealed a similar defect in ADPr removal in ARH3 KO cells and patient fibroblast (eFigure 2A). Given that ARH3 is believed to preferentially hydrolyse mono-ADPr (MAR) modifications, we repeated the experiment using a mono-ADPr-specific reagent, confirming a functional defect in MAR removal in ARH3 KO and patient fibroblast cells (eFigure 2B).

Similar results were obtained by immunofluorescence staining, with a slight, but statistically significant, increase in basal MAR/PAR signal in ARH3 KO cells compared with controls and a reproducible, but not statistically significant, increase in patient fibroblasts compared with their controls. As with the Western blotting experiments above, a clear defect in ADPr removal 1 hour after recovery from hydrogen peroxide treatment was observed in both sets of ARH3-deficient cells (Figure 2B).

These data strongly indicate that the patient cells are indeed deficient in ARH3 function, in a manner similar to ARH3 KO cells, leading to the accumulation of ADP-ribosylated proteins, in particular histones.

### Minocycline Is a Poor PARP1/2 Inhibitor

**Figure 3:**
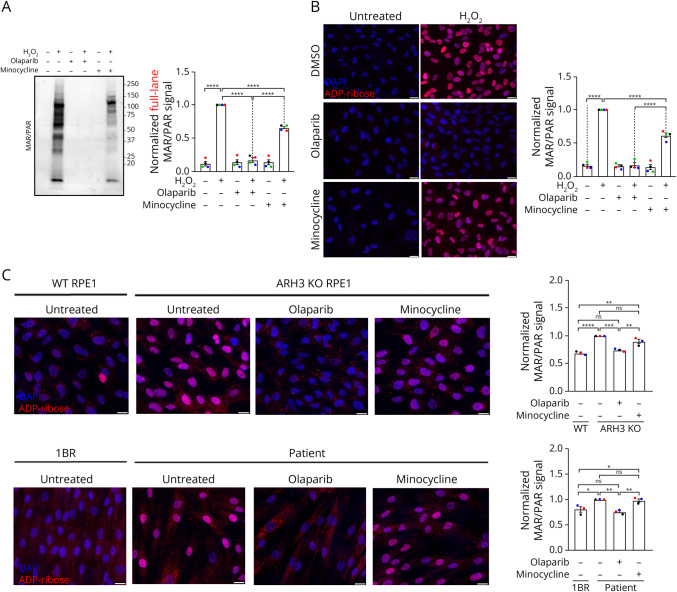
Minocycline Is a Poor PARP1/2 Inhibitor (A) Representative image (left) and quantification (right) of pan-ADP-ribose (MAR/PAR) immunoblots from WT RPE1 cells treated or not with H_2_O_2_ (600 µM, 10 minutes) and DMSO, 10 µM olaparib, or 10 µM minocycline. Full-lane signal was quantified. (B) Representative images (left) and quantification (right) of pan-ADP-ribose (MAR/PAR) immunofluorescence assay after the same treatment conditions as in A. Nuclei were stained with DAPI (blue) or pan-ADP-ribose reagent (red). The mean MAR/PAR intensity within each nucleus was quantified, and the values for thousands of nuclei were averaged for each replicate/sample. For normalization, the signal for H_2_O_2_-treated controls in each replicate was arbitrarily defined as 1. Scale bars = 50 µm (C) Representative images (left) and quantification (right) of pan-ADP-ribose (MAR/PAR) immunofluorescence assay in WT and ARH3 KO RPE1 cells (top) or in 1BR and patient fibroblasts (bottom), comparing untreated cells with cells treated for 24 hours with 10 µM olaparib or 10 µM minocycline, as indicated. The mean MAR/PAR intensity within each nucleus was quantified, and the values for thousands of nuclei were averaged for each replicate/sample. For normalization, the signal of untreated ARH3 KO RPE1 cells or patient fibroblasts in each replicate was arbitrarily defined as 1. Scale bars = 20 µm; mean and SEM of 4 (A and B) or 3 (C) independent experiments. The one-way ANOVA and Tukey test were performed. ns = not significant, * = *p* < 0.05, ** = *p* < 0.005, *** = *p* < 0.0005, and **** = *p* < 0.0001.

The antibiotic minocycline has been suggested as a potential treatment for patients with CONDSIAS because of its proposed activity as a PARP1 inhibitor.^22,36,37^ To test its effectiveness, we first treated RPE1 cells with minocycline and olaparib, a clinically relevant PARP1/2 inhibitor,^38^ and exposed these cells to hydrogen peroxide as in the above experiments. For these experiments, both olaparib and minocycline were used at 10 µM, which is close to the reported peak plasma concentration of both compounds.^39,40^Using Western blotting to quantify protein ADP-ribosylation, we observed that minocycline slightly reduced H_2_O_2_-induced MAR/PAR signal but was unable to completely inhibit PARP1/2 activity to the extent that olaparib did (Figure 3A). To corroborate this finding, we repeated the same experiment, quantifying MAR/PAR by immunofluorescence staining (Figure 3B). Again, minocycline only partially reduced peroxide-induced ADP-ribosylation, while olaparib completely prevented MAR/PAR formation.

Given that ARH3-deficient cells accumulate ADP-ribosylated proteins under basal conditions (Figure 2, A and B), and that long-term PARP1/2 inhibition with olaparib prevented the accumulation of these chromatin “scars” in these cells,^19^ we tested whether long-term minocycline treatment would similarly suppress the accumulation of basal ADPr in ARH3 KO and/or patient fibroblast cells. Using immunofluorescence staining, we observed the expected increase in basal MAR/PAR signal in the nuclei of ARH3 KO cells and patient fibroblasts compared with their respective controls and confirmed that a 24-hour treatment with olaparib completely suppressed the formation of this signal (Figure 3C), as previously reporte.^19^ However, a 24-hour treatment with 10 µM minocycline had no appreciable effect on basal ADPr, suggesting that minocycline is not an effective PARP1/2 inhibitor.

## Discussion

CONDSIAS is a rare genetic disorder caused by biallelic loss-of-function variants in *ADPRS*. Since its first description in 2018,^12,13^ several additional pathogenic variants have been described.^15-18,41-47^ Here, we present the first documented case of CONDSIAS in a South American patient, presenting compound heterozygous variants (p.Q106* and p.Leu162Pro) in the gene encoding ARH3. We provide evidence that c.485T > C; p.Leu162Pro is a novel pathogenic *ADPRS* variant that causes destabilization of ARH3 protein, as evidenced by a severe reduction in ARH3 protein levels in patient fibroblasts (Figure 1). Modeling data indicated the possibility that the variant protein is misfolded, but we found no evidence of degradation by the proteasome. Given the strong effect of this variant on ARH3 protein stability, we cannot draw any conclusions regarding a direct effect of the Leu162Pro substitution on ARH3 enzymatic activity. Nonetheless, the data presented here allow the classification of this variant as pathogenic, according to ACMG/AMP criteria (based on criteria PS3, PM2, PM3, and PP4).^34^

Our results strongly indicate that patient fibroblasts are defective in ARH3 enzymatic activity because they displayed a clear defect in the hydrolysis of hydrogen peroxide-induced ADP-ribosylation, to a similar extent as CRISPR/Cas9-generated ARH3 KO cells (Figure 2). Perhaps more relevant to CONDSIAS disease pathology, patient fibroblasts also accumulated higher levels of endogenous ADP-ribosylation under basal growth conditions (Figure 2 and 3C). As previously reported, ADPr accumulation in these cells occurred predominantly on low molecular weight proteins, which are likely to be histones. Given that histone H3 serine 10 is a major acceptor site for DNA damage–induced ADP-ribosylation,^48^ we speculate that patient cells are accumulating unremoved mono-ADP-ribose “scars” on chromatin, which have been shown to cause transcription deregulation by interfering with H3 lysine 9 acetylation (H3K9ac), an important epigenetic modification.^19,49^

PARP1/2 inhibitors, which are in clinical use in oncology, have been proposed as potentially beneficial for CONDSIAS patients, because the pathology is likely to arise from the accumulation of mono-ADP-ribosylated proteins,^19^ but these compounds are not regulated for use in this setting. The potential benefits, risks, and difficulties of repurposing PARP1 inhibitors for nononcological disease have been reviewed for several pathologies associated with PARP activity.^37^ In the absence of comprehensively tested therapeutic strategies, some CONDSIAS patients, including the patient presented here, are reported to be making use of minocycline,^17,21^ an antibiotic of the tetracycline family, which is readily available for prescription by clinicians, and was shown to have PARP1 inhibitory activity in the past.^22^ Given this clinical interest, we revisited this question using the improved ADPr detection reagents developed over the last several years, which were not available when the PARP1 inhibition by minocycline was first reported almost 20 years ago.^22^ In agreement with other studies,^50^ we observed only a partial reduction in hydrogen peroxide-induced ADP-ribosylation in cells treated with minocycline. It is worth mentioning that the 10 µM minocycline concentration used here is similar to peak plasma concentration of the drug in humans^40^ and that this concentration is almost 1000-fold above the reported IC_50_ of 12.5 nM for PARP1 inhibition by minocycline.^22^ While the partial effect we observed could be ascribed to inefficient PARP1 inhibition by minocycline in cells, another possibility, which we favor, is that minocycline slightly reduces PARP1/2 activation in these experiments because of its known antioxidant activities,^50,51^ which would reduce the formation of single-strand breaks required for PARP1 activation. In agreement with this idea, minocycline treatment was unable to reduce the accumulation of basal ADP-ribosylated proteins in ARH3 KO or patient fibroblasts (Figure 3C). The latter result is particularly important for the molecular rationale of using minocycline therapy in CONDSIAS because the basal accumulation of ADPr is believed to underpin disease pathology.^19^

In conclusion, we present a novel causative variant for CONDSIAS and caution against minocycline use for management of this disorder, arguing in favor of further studies investigating the clinical benefits of potent PARP inhibitors for the treatment of this pathology.

## Glossary

ADPr: ADP-ribosylation
ARH3: ADP-ribosyl hydrolase 3
ASD: autism spectrum disorder
PAR: poly-ADP-ribose
PARG: poly-ADP-ribose glycohydrolase
PARPs: poly-ADP-ribose polymerases
